# Assessment of Hospital Readmission Rates, Risk Factors, and Causes After Cardiac Arrest: Analysis of the US Nationwide Readmissions Database

**DOI:** 10.1001/jamanetworkopen.2019.12208

**Published:** 2019-09-27

**Authors:** Ilhwan Yeo, Jim W. Cheung, Dmitriy N. Feldman, Nivee Amin, John Chae, S. Chiu Wong, Luke K. Kim

**Affiliations:** 1 Division of Hospital Medicine, Department of Medicine, Icahn School of Medicine at Mount Sinai, The Mount Sinai Hospital, New York, New York; 2 Weill Cornell Cardiovascular Outcomes Research Group, Division of Cardiology, Department of Medicine, Weill Cornell Medicine, New York Presbyterian Hospital, New York; 3 Weill Cornell Medicine, New York Presbyterian Hospital, New York

## Abstract

This cohort study investigates the rate, timing, and causes of hospital readmission after cardiac arrest and the risk factors associated with readmission.

## Introduction

Cardiac arrest (CA) remains a global health challenge with high rates of mortality and morbidity.^1,2^ Furthermore, recovery from CA without residual neurologic deficit is limited. Consequently, the burden of CA on the US health care system is increasing.

Thirty-day readmissions are costly and associated with poor outcomes.^3^ However, there is a paucity of data regarding the readmission characteristics of CA, and previous studies have mostly focused on older populations.^4^ Therefore, further understanding of readmission after CA is needed to allow institutions to focus already limited resources and prevent unnecessary readmissions. We aimed to investigate contemporary rate, timing, causes, and risk factors associated with 30-day readmissions after CA.

## Methods

This cohort study used data from the Nationwide Readmissions Database (NRD) from 2010 to 2014. Data analysis was performed from January 1, 2010, to November 30, 2014. The NRD collects annual discharge data and enables nationally representative readmission analyses.^5^ All hospitalizations associated with either out-of-hospital CA or in-hospital CA were selected based on the *International Classification of Diseases, Ninth Revision, Clinical Modification* code 427.5. Among those with CA, ventricular tachycardia and ventricular fibrillation were identified by codes 427.1 and 427.4, respectively. Pulseless electrical activity or asystole arrests were defined as CA without concomitant ventricular arrhythmia. The primary outcome of interest was 30-day all-cause readmission. To identify independent risk factors associated with 30-day readmission following discharge after CA, we created a multivariable Cox proportional hazards regression model. The Weill Cornell Medicine institutional review board deemed this study exempt because the NRD is a publicly available database containing deidentified patient information. All analyses were performed using SAS statistical software version 9.4 (SAS Institute). All tests were 2-sided, with *P* < .05 indicating statistical significance. This study followed the Strengthening the Reporting of Observational Studies in Epidemiology (STROBE) reporting guideline.

## Results

There were 251 346 patients who survived the CA-related index hospitalization. Median (interquartile range) age was 64.8 (53.7-75.8) years, and 106 831 participants (42.5%) were women (Table 1). Among CA survivors, 49 305 (19.6%) were readmitted within 30 days after discharge. While 30-day readmission rate was higher in the cohort with pulseless electrical activity or asystole than in the cohort with ventricular tachycardia or ventricular fibrillation (20.3% vs 18.3%; difference, 2.0%; 95% CI, 1.7%-2.4%; *P* < .001), the median (interquartile range) time to readmission was 9 (4-18) days for both cohorts.

**Table 1.** Baseline Individual- and Hospital-Level Characteristics for Cardiac Arrest Survivors Stratified by Causative Rhythm

Characteristic	No. (%)						
	Overall	30-Day Readmission					
		VT or VF (n = 84 854)			PEA or Asystole (n = 166 492)		
		No	Yes	*P* Value	No	Yes	*P* Value
No. of patients	251 346	69 358 (81.7)	15 496 (18.3)		132 683 (79.7)	33 809 (20.3)	
Age, median (IQR), y	64.8 (53.7-75.8)	62.6 (52.8-72.8)	65.2 (55.2-74.9)	<.001	66.1 (54.3-77.2)	66.4 (55.6-76.8)	.42
Female	106 831 (42.5)	23 152 (33.4)	5682 (36.7)	<.001	61 572 (46.4)	16 425 (48.6)	<.001
ST-elevation myocardial infarction	32 584 (13.0)	19 125 (27.6)	3368 (21.7)	<.001	8295 (6.3)	1796 (5.3)	<.001
Pulmonary embolism	8563 (3.4)	1403 (2.0)	393 (2.5)	.07	5423 (4.1)	1344 (4.0)	.62
Coma	9790 (3.9)	2942 (4.2)	486 (3.1)	<.001	5328 (4.0)	1034 (3.1)	<.001
Hypertension	153 419 (61.0)	41 415 (59.7)	9613 (62.0)	.004	80 500 (60.7)	21 890 (64.7)	<.001
Diabetes	83 635 (33.3)	19 518 (28.1)	5502 (35.5)	<.001	44 589 (33.6)	14 026 (41.5)	<.001
Coronary artery disease	104 277 (41.5)	40 067 (57.8)	8812 (56.9)	.26	43 323 (32.7)	12 075 (35.7)	<.001
Myocardial infarction	19 831 (7.9)	7326 (10.6)	1631 (10.5)	.94	8424 (6.3)	2450 (7.2)	<.001
Percutaneous coronary intervention	17 686 (7.0)	6350 (9.2)	1390 (9.0)	.70	7794 (5.9)	2153 (6.4)	.08
Coronary artery bypass graft	16 437 (6.5)	4908 (7.1)	1204 (7.8)	.11	8090 (6.1)	2234 (6.6)	.07
Congestive heart failure	100 016 (39.8)	30 327 (43.7)	8760 (56.5)	<.001	45 980 (34.7)	14947 (44.2)	<.001
Cardiac arrest	5523 (2.2)	2378 (3.4)	379 (2.4)	<.001	2315 (1.7)	451 (1.3)	.002
Peripheral vascular disease	20 980 (8.3)	4977 (7.2)	1608 (10.4)	<.001	10 792 (8.1)	3603 (10.7)	<.001
Pulmonary hypertension	18 339 (7.3)	4446 (6.4)	1384 (8.9)	<.001	9562 (7.2)	2947 (8.7)	<.001
Chronic pulmonary disease	49 776 (19.8)	11 565 (16.7)	3377 (21.8)	<.001	26 517 (20.0)	8318 (24.6)	<.001
Chronic kidney disease	49 755 (19.8)	11 057 (15.9)	3657 (23.6)	<.001	26 303 (19.8)	8737 (25.8)	<.001
Hemodialysis	26 341 (10.5)	4509 (6.5)	2160 (13.9)	<.001	13 720 (10.3)	5951 (17.6)	<.001
Anemia	71 400 (28.4)	16 135 (23.3)	4696 (30.3)	<.001	38 499 (29.0)	12 070 (35.7)	<.001
Atrial fibrillation	65 876 (26.2)	18 934 (27.3)	4792 (30.9)	<.001	32 881 (24.8)	9269 (27.4)	<.001
Coagulopathy	36 886 (14.7)	9171 (13.2)	2506 (16.2)	<.001	19 516 (14.7)	5694 (16.8)	<.001
Obesity	37 946 (15.1)	10 306 (14.9)	2425 (15.7)	.18	19 677 (14.8)	5537 (16.4)	<.001
Pulmonary circulation disorders	15 909 (6.3)	2343 (3.4)	887 (5.7)	<.001	9676 (7.3)	3003 (8.9)	<.001
Valvular heart disease	15 116 (6.0)	2862 (4.1)	960 (6.2)	<.001	8781 (6.6)	2513 (7.4)	.005
Elixhauser Comorbidity Index score >4	141 903 (56.5)	33 711 (48.6)	9739 (62.8)	<.001	75 425 (56.8)	23 028 (68.1)	<.001
Procedures performed							
Coronary angiography	76 621 (30.5)	40 815 (58.8)	7632 (49.3)	<.001	22 684 (17.1)	5489 (16.2)	.04
Percutaneous coronary intervention	36 994 (14.7)	21 626 (31.2)	3958 (25.5)	<.001	9341 (7.0)	2070 (6.1)	.002
Intra-aortic balloon pump	13 546 (5.4)	7062 (10.2)	1647 (10.6)	.41	3753 (2.8)	1084 (3.2)	.06
Percutaneous left ventricular assist device	998 (0.4)	504 (0.7)	147 (0.9)	.10	288 (0.2)	59 (0.2)	.38
Targeted temperature management	7190 (2.9)	4033 (5.8)	583 (3.8)	<.001	2102 (1.6)	473 (1.4)	.18
Teaching hospital^a^	140 812 (56.0)	40 153 (57.9)	8971 (57.9)	>.99	72 685 (54.8)	19 003 (56.2)	.01
Urban hospital location^b^	137 506 (54.7)	37 559 (54.2)	8931 (57.6)	<.001	71 478 (53.9)	19 538 (57.8)	<.001
Length of hospital stay, median (IQR), d	10.1 (4.8-19.6)	8.8 (4.6-16.3)	12.2 (6.5-22.0)	<.001	9.8 (4.4-19.8)	13.7 (7.0-24.7)	<.001
Prolonged hospital stay^c^	65 184 (25.9)	13 563 (19.6)	4799 (31.0)	<.001	34 842 (26.3)	11 979 (35.4)	<.001

Abbreviations: IQR, interquartile range; PEA, pulseless electrical activity; VF, ventricular fibrillation or flutter; VT, ventricular tachycardia.

^a^ Nonteaching hospital as reference.

^b^ Rural hospital location as reference.

^c^ Length of stay days exceeding the 75th percentile (≥20 days) of the entire stay days.

Overall, approximately three-quarters (72.1%) of the 30-day readmissions were due to noncardiac causes, which were more common among patients with pulseless electrical activity or asystole than those with ventricular tachycardia or ventricular fibrillation (77.2% vs 61.4%; difference, 15.7%; 95% CI, 14.9%-16.6%; *P* < .001). Among noncardiac causes, infectious etiology (pneumonia and sepsis) was most prevalent (18.9%), followed by chronic obstructive pulmonary disease or respiratory failure (13.3%). Heart failure and arrhythmia accounted for more than 50% of all cardiac causes of readmission. After adjusting for baseline characteristics, several comorbidities were independently associated with a higher risk of 30-day readmission across the rhythm cohorts (Table 2).

**Table 2.** Risk Factors Associated With 30-Day Readmission After Cardiac Arrest–Related Index Hospitalization

Covariate	VT or VF				PEA or Asystole			
	Univariate^a^		Multivariable^b^		Univariate^a^		Multivariable^b^	
	Unadjusted HR (95% CI)	*P* Value	Adjusted HR (95% CI)	*P* Value	Unadjusted HR (95% CI)	*P* Value	Adjusted HR (95% CI)	*P* Value
Female	1.14 (1.07-1.21)	<.001	1.05 (0.99-1.12)	.13	1.08 (1.04-1.12)	<.001	1.06 (1.02-1.11)	.002
Chronic kidney disease receiving hemodialysis	2.07 (1.92-2.24)	<.001	1.56 (1.43-1.70)	<.001	1.70 (1.62-1.78)	<.001	1.44 (1.36-1.52)	<.001
Prolonged hospital stay^c^	1.71 (1.61-1.81)	<.001	1.38 (1.30-1.48)	<.001	1.46 (1.40-1.52)	<.001	1.35 (1.29-1.41)	<.001
History of congestive heart failure	1.58 (1.49-1.68)	<.001	1.27 (1.20-1.36)	<.001	1.42 (1.37-1.48)	<.001	1.19 (1.14-1.24)	<.001
Chronic kidney disease	1.53 (1.43-1.64)	<.001	1.21 (1.12-1.30)	<.001	1.35 (1.29-1.41)	<.001	1.17 (1.12-1.23)	<.001
Chronic pulmonary disease	1.34 (1.25-1.43)	<.001	1.16 (1.08-1.25)	<.001	1.27 (1.21-1.33)	<.001	1.18 (1.12-1.24)	<.001
Intra-aortic balloon pump	1.04 (0.94-1.16)	.41	NC^d^	NC	1.13 (1.00-1.27)	.048	1.18 (1.04-1.34)	.01
Peripheral vascular disease	1.43 (1.30-1.56)	<.001	1.16 (1.06-1.27)	.002	1.29 (1.21-1.38)	<.001	1.11 (1.03-1.19)	.005
Percutaneous coronary intervention	0.78 (0.73-0.83)	<.001	1.13 (1.03-1.24)	.007	0.88 (0.81-0.95)	.002	0.97 (0.87-1.08)	.57
Diabetes	1.35 (1.27-1.43)	<.001	1.08 (1.01-1.15)	.02	1.34 (1.29-1.40)	<.001	1.12 (1.07-1.17)	<.001
Anemia	1.37 (1.29-1.46)	<.001	1.07 (1.00-1.14)	.06	1.31 (1.26-1.37)	<.001	1.06 (1.02-1.11)	.007
Urban hospital location^e^	1.14 (1.07-1.21)	<.001	1.08 (1.01-1.15)	.02	1.15 (1.10-1.20)	<.001	1.06 (1.01-1.10)	.009
Elixhauser Comorbidity Index score >4	1.68 (1.58-1.79)	<.001	1.06 (0.97-1.15)	.24	1.54 (1.48-1.60)	<.001	1.06 (1.00-1.13)	.04
Atrial fibrillation	1.17 (1.10-1.24)	<.001	1.01 (0.95-1.07)	.79	1.13 (1.08-1.18)	<.001	1.05 (1.01-1.10)	.03
Hypertension	1.09 (1.03-1.16)	.003	0.93 (0.87-0.99)	.02	1.17 (1.12-1.22)	<.001	0.99 (0.94-1.03)	.50
Coronary angiography	0.71 (0.67-0.75)	<.001	0.89 (0.83-0.95)	<.001	0.95 (0.90-1.00)	.04	0.98 (0.92-1.05)	.61
History of cardiac arrest	0.72 (0.61-0.85)	<.001	0.81 (0.69-0.96)	.01	0.78 (0.67-0.92)	.003	0.77 (0.65-0.90)	.001
Targeted temperature management	0.66 (0.57-0.77)	<.001	0.76 (0.66-0.89)	<.001	0.90 (0.76-1.07)	.22	NC	NC
Coma	0.76 (0.65-0.89)	<.001	0.73 (0.62-0.84)	<.001	0.78 (0.70-0.86)	<.001	0.75 (0.67-0.82)	<.001

Abbreviations: HR, hazard ratio; NC, not calculated; PEA, pulseless electrical activity; VF, ventricular fibrillation or flutter; VT, ventricular tachycardia.

^a^ Univariate Cox proportional hazards regression model was created with an outcome of 30-day readmission for each covariate from Table 1.

^b^ Multivariable Cox proportional hazards regression model was created with an outcome of 30-day readmission including all covariates with *P* < .10 in the univariate analysis, and the covariates with *P* < .05 for either rhythm cohort are listed.

^c^ Length of stay days exceeding the 75th percentile (≥20 days) of the entire stay days.

^d^ Covariate with *P* ≥ .10 in the univariate analysis was not included in the multivariable analysis.

^e^ Rural hospital location as reference.

## Discussion

Given the high readmission rates and substantial economic burden associated with CA, nationwide efforts are necessary to develop strategies designed explicitly for CA survivors to reduce preventable readmissions. Of those readmitted within 30 days, more than half were readmitted within 9 days, especially for noncardiac causes. Close outpatient follow-up during the first 10 days after hospitalization may be an opportunity for clinicians to preemptively intervene on any evolving medical conditions and consequently prevent readmissions for CA survivors.^6^ Furthermore, patients with limited access to health care owing to their socioeconomic status have been shown to use the emergency department more as a primary source of care, which may lead to more readmissions. Therefore, multidisciplinary efforts to support the transition from inpatient to outpatient care with a readily available support system, including proper patient education, follow-up telephone calls, use of remote telemonitoring, clinician home visits, and postdischarge hotlines are potential strategies to consider. A limitation of our study is that we were unable to validate the codes for comorbidities from the *International Classification of Diseases, Ninth Revision, Clinical Modification*.

## Conclusions

This cohort study found increased rates of readmission among patients who survived CA. Early follow-up with health care professionals may enable timely management of both cardiac and general medical conditions and reduce preventable readmissions of CA survivors.
